# Parameter, noise, and tree topology effects in tumor phylogeny inference

**DOI:** 10.1186/s12920-019-0626-0

**Published:** 2019-12-23

**Authors:** Kiran Tomlinson, Layla Oesper

**Affiliations:** 1 Department of Computer Science, Carleton College, 1 N College St, Northfield, 55057 MN USA; 2 Department of Computer Science, Cornell University, 402 Gates Hall, Ithaca, 14853 NY USA

**Keywords:** Cancer genomics, Tumor phylogeny, Evolution

## Abstract

**Background:**

Accurate inference of the evolutionary history of a tumor has important implications for understanding and potentially treating the disease. While a number of methods have been proposed to reconstruct the evolutionary history of a tumor from DNA sequencing data, it is not clear how aspects of the sequencing data and tumor itself affect these reconstructions.

**Methods:**

We investigate when and how well these histories can be reconstructed from multi-sample bulk sequencing data when considering only single nucleotide variants (SNVs). Specifically, we examine the space of all possible tumor phylogenies under the infinite sites assumption (ISA) using several approaches for enumerating phylogenies consistent with the sequencing data.

**Results:**

On noisy simulated data, we find that the ISA is often violated and that low coverage and high noise make it more difficult to identify phylogenies. Additionally, we find that evolutionary trees with branching topologies are easier to reconstruct accurately. We also apply our reconstruction methods to both chronic lymphocytic leukemia and clear cell renal cell carcinoma datasets and confirm that ISA violations are common in practice, especially in lower-coverage sequencing data. Nonetheless, we show that an ISA-based approach can be relaxed to produce high-quality phylogenies.

**Conclusions:**

Consideration of practical aspects of sequencing data such as coverage or the model of tumor evolution (branching, linear, etc.) is essential to effectively using the output of tumor phylogeny inference methods. Additionally, these factors should be considered in the development of new inference methods.

## Background

Cancer is caused by somatic mutations in a single founder cell that lead to the unrestrained proliferation of the descendants of that cell. According to the clonal theory of cancer [1], descendants of the founder cell will continue to acquire new somatic mutations that may drive disease progression. Since different descendants acquire distinct mutations, the history of a tumor can be described as a type of phylogenetic tree. In these trees, vertices represent tumor cell populations, or clones, each with their own set of somatic mutations, and edges represent ancestral relationships between populations. Several different models of tumor evolution have been proposed, including linear, branching, neutral, and punctuated evolution [2–4], describing different patterns of how and when new tumor populations arise. As a result of these evolutionary processes, a tumor itself may be a heterogeneous mix of different tumor cell populations.

A number of recent studies have highlighted the prevalence of such *intra-tumor heterogeneity* [5–7] across many different cancer types. Computational methods for analyzing intra-tumor heterogeneity, including characterization of the populations in a particular tumor and how they evolved, have important implications for understanding and, ultimately, treating the disease [8, 9]. For example, cancer types that are typically detected late in the tumor’s evolution, such as pancreatic cancer, often have poor prognosis [10]. Intra-tumor heterogeneity may play a key role in therapeutic failure in such instances if the treatment only targets certain tumor cell populations [11]. Treatment strategies that take the evolutionary history of a tumor into account by specifically targeting clonal mutations (those present in every tumor cell) [12] or that combine drugs based on a patient’s specific tumor evolutionary history [13] have the potential to be more effective. However, for such approaches to be feasible, there is an imperative need for better approaches to inferring and analyzing the evolutionary history of a single tumor.

There has been an increased recent interest in computational methods that use noisy DNA sequencing data to reconstruct the evolutionary history of a tumor in terms of ancestral relationships between somatic mutations. A number of recent approaches have focused on using single-cell sequencing data to reconstruct tumor phylogenies [14–16]. Ultimately, such methods have the promise to provide improved resolution for such reconstructions. However, currently single-cell sequencing still suffers from both high error rates and high cost. While technological and methodological developments are beginning to alleviate these issues, the majority of the currently available data is still from bulk sequencing experiments. Specifically, most large scale cancer studies such as The Cancer Genome Atlas (TCGA) and the International Cancer Genome Consortium (ICGC) have made this type of data widely available. Thus, there is still much to be gained from methods that analyze bulk data, while single-cell methods continue to mature. Therefore, we focus here on the data from more economical bulk sequencing. However, there are still many challenges and sources of error in this type of data. In bulk sequencing, collections of potentially heterogeneous cells are sequenced together, which obfuscates the coincidence of mutations. Sources of error include the sequencing process, read alignment, and variant calling algorithms. Thus, specialized methods are required to robustly analyze noisy bulk sequencing data.

Many recent computational methods have been developed to infer tumor phylogenetic trees using multi-sample bulk sequencing data. A large fraction of these methods consider only single nucleotide variants (SNVs) [17–21] and use rules regarding the observed frequencies of each such mutation to identify possible ancestral relationships. In particular, these methods use the infinite sites assumption (ISA), which states that any locus in the genome mutates at most once during the history of tumor, a simplification that makes the underlying computational problem more tractable. For example, AncesTree [17] constructs a graph called the ancestry graph using mutation frequencies and then finds spanning trees of that graph adhering to the ISA. However, increasing reports that the ISA is often violated in cancer [22] have led to the development of methods that relax the ISA in some contexts [16, 23]. Some methods also consider structural variants or copy number aberrations [24–27] in addition to SNVs, but this has proven challenging. Finally, several methods allow for multiple tumor evolutionary trees consistent with a given sequencing dataset by enumerating these trees [18, 26, 27]. Along these lines, a recent paper [28] observed that multiple such trees typically exist in noise free simulations. However, it is unclear how the conclusions from that work are affected by the variety of sources of noise present in bulk sequencing data and to what extent these conclusions apply to real sequencing data. Finally, it is not obvious how existing tumor phylogeny inference methods are affected by the distinct tree topologies resulting from different models of tumor evolution such as branching or linear [2].

In this paper, we investigate several extensions to the ancestry graph approach of [17], which relies on the ISA, and quantify when and how well this approach can reconstruct tumor evolutionary histories from multi-sample bulk sequencing data. In particular, we focus on the performance of this method when applied to noisy data. Our specific methodological contributions include: (1) a relaxation of the ancestry graph approach that makes it more robust to noise; and (2) a method for simplifying the ancestry graph that leads to reduced computational costs. Furthermore, our contributions include extensive analysis of the effects of coverage, noise, evolutionary tree topology, and other parameters in reconstructing clonal trees in simulated data. This analysis has numerous potential future implications for both experimental design and algorithm development. Finally, we apply our methods to cancer sequencing datasets from two studies [29, 30].

## Methods

This section is organized as follows. We begin by outlining the existing ancestry graph method [17] and then formalize the new problem of using this method to enumerate all tumor phylogenies consistent with a particular dataset. We then describe a relaxation that improves the method’s robustness to noise, and introduce a graph simplification that reduces computational cost. Finally, we describe our data simulation procedure and our tree evaluation metric.

### Problem Formalization

#### Definitions

We use *s* to denote the number of samples sequenced from a tumor and *n* to denote the number of mutations observed across all samples. We label these mutations 1,…,*n*. The *s*×*nvariant allele frequency (VAF) matrixF* stores in entry *F*_*ij*_ the fraction of reads from sample *i* containing mutation *j*. A *clonal treeT* (or tumor phylogeny) is a rooted tree on *n* nodes with each node labeled by a distinct mutation. Nodes may also be labeled with disjoint sets of mutations, with a corresponding decrease in the number of nodes. Each node represents a tumor cell population that contains all mutations along its root-node path. The infinite sites assumption (ISA) guarantees that a clonal tree is a perfect phylogeny where mutations evolve without homoplasy. Because of this, we can also represent the tree as an *n*×*nclonal matrixB*, in which *B*_*ℓj*_=1 if cell population *ℓ* contains mutation *j* and 0 otherwise. Finally, the *s*×*nusage matrixU* stores in *U*_*iℓ*_ the proportion of cells in sample *i* that belong to population *ℓ*.

#### The VAFFP and the Ancestry Graph

The authors of [17] formalized the Variant Allele Frequency Factorization Problem (VAFFP), also called the Perfect Phylogeny Mixture Problem in [28], as follows:

*Given*: A VAF matrix *F*.

*Find:* A usage matrix *U* and a clonal matrix *B* such that:

1 $$ F = \frac{1}{2} U B.  $$

The 1/2 factor appears because we assume that all mutations are heterozygous SNVs (implicitly assuming no copy number aberrations). The VAFFP has been shown to be NP-complete [17], but in practice, many datasets are small enough that finding solutions is feasible.

**Fig. 1:**
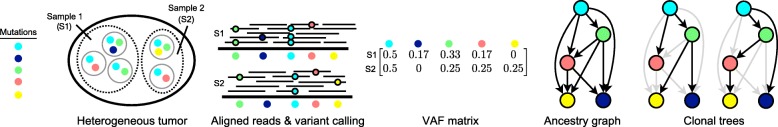
Overview of the clonal tree inference process. From left to right: multiple samples are taken from a heterogeneous tumor, either from different anatomical sites or different times; the samples are sequenced, the resulting reads are aligned to a reference genome, and variants are called; the VAF matrix is built from the reference and variant read counts; we build an ancestry graph from the VAF matrix; each ancestry graph spanning tree that adheres to the sum condition is a candidate clonal tree, two of which are shown. Notice that the second tree could be discounted if we were aware of mutation co-occurrence, because the dark blue and green mutations always appear together in the tumor

The authors of [17] describe a method for solving the VAFFP using the *ancestry graph* of *F* (see Fig. 1 for a visual overview of this approach). In order to avoid confusion, we will often refer to the ancestry graph as the *strict* ancestry graph. The ancestry graph *G*_*F*_ contains *n* nodes, one labeled by each mutation. Additionally, *G*_*F*_ includes a directed edge from node *j* to node *k* if *F*_*ij*_≥*F*_*ik*_ ∀*i*∈{1,…,*s*}. These edges encode the *ancestry condition*: under the ISA, an ancestral mutation must be more frequent than a descendant mutation. The possible clonal trees are exactly the set of directed spanning trees of *G*_*F*_ that adhere to the *sum condition* (2). Using *C*(*j*) to denote the children of mutation *j* in a clonal tree *T*, the sum condition requires that:

2 $$  \sum_{k \in C(j)} F_{ik} \le F_{ij} \qquad \forall i \in \{1, \dots, s\}.  $$

That is, the sum of observed frequencies of sibling mutations in a clonal tree cannot exceed the frequency of their parent mutation in any sample.

Every spanning tree *T* of *G*_*F*_ that adheres to the sum condition corresponds to a VAFFP solution (see the rightmost part of Fig. 1 for examples). The clonal matrix *B* can be constructed from *T* by tracing through each root-leaf path in *T*. The matrix *U* can be efficiently computed using the following equation from [17]:

3 $$ U_{ij} = 2\Big(F_{ij} - \sum_{k \in C(j)}F_{ik} \Big).  $$

#### The Enumeration Variant Allele Frequency Factorization Problem (E-VAFFP)

Here, we define the focus of our work, the enumeration version of the VAFFP.

*Given*: A VAF matrix *F*.

*Find*: The set $\mathcal {T}(G_{F})$ of *all* trees that span the ancestry graph *G*_*F*_ and adhere to the sum condition.

We say that an E-VAFFP solution *exists* or that *Fadmits an E-VAFFP solution* when $\mathcal {T}(G_{F}) \ne \emptyset $. In this paper, we explore the relationship between $\mathcal {T}(G_{F})$ and the underlying tumor evolutionary tree, and present several relaxations and extensions to the E-VAFFP.

### Finding and Counting E-VAFFP Solutions

In order to solve the E-VAFFP, we employ a modified version of the Gabow-Myers algorithm [31]. Specifically, this algorithm uses a structured depth-first search in order to recursively construct all spanning trees of the graph. It is straightforward to modify this approach to avoid execution branches that violate the sum condition, as has been done previously by [19, 26, 28]. Additionally, we note that the number of such spanning trees of *G*_*F*_ is the product of its non-root in-degrees [28, 32]. This provides an upper bound on $|\mathcal {T}(G_{F})|$.

### Relaxing the E-VAFFP

#### Approximate Ancestry Graph

Real DNA sequencing data is often quite noisy, but the E-VAFFP assumes that *F* is measured exactly. In real data, *G*_*F*_ often has no spanning trees. To handle less idealized data, we use a method based on the probabilistic approach from [17]. This approach defines the *approximate ancestry graph* of *F*: a complete *n*-node directed graph with nodes labeled by mutations and edges (*j*,*k*) weighted by the probability that mutation *j* is ancestral to mutation *k* given their observed frequencies. To calculate this probability, we model reads as being drawn from a binomial distribution with a flat prior on the proportion parameter. Thus, we model the resulting posterior distribution for the VAF of mutation *j* in sample *i* with observed variant and reference read counts *v*_*ij*_ and *r*_*ij*_, respectively, with the beta-distributed random variable *X*_*ij*_∼*Beta*(*v*_*ij*_+1,*r*_*ij*_+1), as done in [17]. If *X*_*ij*_≥*X*_*ik*_, then this provides evidence that mutation *j* is ancestral to mutation *k*. The overall probability that *j* is ancestral to *k* is defined based on the sample with the weakest evidence:

4 $$ \text{Pr}[j\text{ ancestral to } k] := \min_{i} \text{Pr}[X_{ij} \ge X_{ik}]  $$

The probabilities on the right hand side of (4) can be calculated from the read counts that generate *F* using the approach described in [33], as both of the random variables *X*_*ij*_ and *X*_*ik*_ are beta-distributed.

Just as we did in the strict ancestry graph, we can also use the Gabow-Myers algorithm [31] to enumerate all spanning trees of the approximate ancestry graph whose observed frequencies satisfy the sum condition. In this context, we refer to such trees as *valid* spanning trees. Once these are computed, we can then select the most probable (i.e. max weight) tree. Alternatively, if the graph has too many spanning trees to fully enumerate, we can use the algorithm of [34] to list weighted spanning trees in descending weight order until we find one satisfying the sum condition. Unlike Gabow-Myers, this algorithm is not easy to modify to include the sum condition. Using this method, we can potentially find the most probable clonal tree without the need to enumerate every tree. However, this approach may be significantly slower when no valid spanning trees exist as the method is forced to explore the entire space of spanning trees rather than just those satisfying the sum condition.

Note that the approximate ancestry graph does not yield more E-VAFFP solutions than the strict ancestry graph. Any tree that violates the sum condition in the strict graph will necessarily violate it in the approximate graph, because the sum condition only depends on the VAF matrix *F*. Additionally, any approximate graph spanning tree not present in the strict graph must violate the ancestry condition (and thus the sum condition), since it includes an edge not present in the strict graph.

Nonetheless, the approximate ancestry graph still provides two key benefits. First, it orders solutions by likelihood, and second, it allows us to explore novel tree topologies not present in the strict graph if we also weaken the sum condition.

#### Relaxed Sum Condition

Adding leniency to the sum condition allows the identification of possible clonal trees rendered invalid by noise. For a small *error threshold* *ε*, we can relax the sum condition to require that:

5 $$  \sum_{k \in C(j)} F_{ik} \le F_{ij} + \varepsilon \qquad \forall i \in \{1, \dots, s\}  $$

We then can identify the smallest *ε* resulting in one valid spanning tree. In other words, we find the spanning tree with the smallest maximal sum condition violation. We note that [19] also relaxes the sum condition in this way, but does not use it in conjunction with an approximate ancestry graph.

### Pruning Transitive Edges

**Fig. 2:**
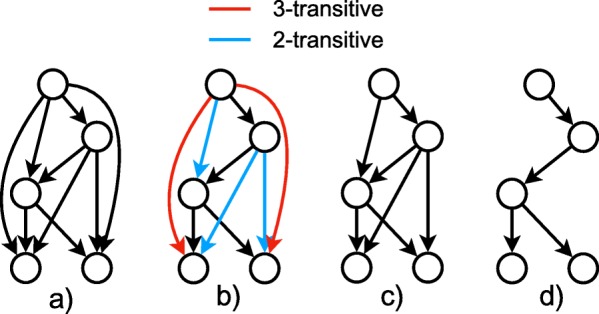
Example of partial transitive reduction. **a** An ancestry graph *G*_*F*_. **b** The transitive edges in *G*_*F*_. The red edges are 3-transitive and the blue edges are 2-transitive. **c** The 3-PTR of *G*_*F*_. **d** The transitive reduction of *G*_*F*_; equivalently, the 2-PTR of *G*_*F*_

The number of spanning trees of an *n*-node DAG grows exponentially with *n* when the average in-degree is held constant. Even with only 20 mutations, the number of spanning trees of *G*_*F*_ can exceed 10^17^, making clonal tree inference extremely slow. We therefore explore the removal of transitive edges from the ancestry graph as a means of reducing the spanning trees present in the graph while maintaining core ancestral relationships (see Fig. 2). This approach may be especially useful if the underlying tumor has a branching rather than a linear topology.

For a directed acyclic graph *G*, we say that an edge (*u*,*v*)∈*G* is *k-transitive* if there is a path from *u* to *v* of length *k* (see Fig. 2b). Additionally, we define an edge to be ≥*k*-transitive if it is *i*-transitive for some *i*≥*k*. By removing all ≥*k*-transitive edges from *G* for a chosen *k*, we can reduce the number of spanning trees while maintaining the general structure of *G*. We call the graph resulting from removing all ≥*k*-transitive edges the *k partial transitive reduction (k-PTR)* of *G*. Note that the 2-PTR is the standard transitive reduction [35] of a graph (see Fig. 2d). To construct the *k*-PTR of *G*, we first find the transitive reduction *R* of *G* using Hsu’s algorithm [36]. Then, we can easily identify if (*u*,*v*) is ≥*k*-transitive by checking the path length from *u* to *v* in *R*. We can do this efficiently by pre-computing the all-pairs shortest path matrix of *R* with *n* breadth-first searches.

### Simulating Noisy VAF Data

We use simulated data to assess our methods. Our data simulation process consists of four steps: (1) randomly generate an evolutionary tree topology, (2) choose the cellular frequencies, (3) determine the mutation frequencies, and (4) draw variant reads from a binomial distribution, allowing direct computation of *F*.

Given the number of mutations *n*, the number of samples *s*, and the average sequencing coverage *c*, we first generate a random tumor phylogeny *T*, referred to as the *underlying tree* for the simulation, and an *s*×*n* VAF matrix consistent with *T*. For simplicity, each clone acquires exactly one new somatic mutation, so we also call *n* the number of clones. We construct *T* iteratively by adding each mutation as the child of a random node already in *T*. From *T*, we compute the clonal matrix *B* described in a previous section. We then generate the cellular frequencies of the *n* clones. Clone *i* is assigned frequency *u*_*i*_ such that $\sum _{i} u_{i} = 1$. To pick *u*_1_,…,*u*_*n*_, we sample uniformly from all possible frequency values using the standard simplex method from [37].

We then calculate the tumor’s mutation frequencies. Using the row vectors $\vec {f}$ and $\vec {u}$ to store mutation and cellular frequencies, respectively, we find $\vec {f}$ using (1):

6 $$ \vec{f} = \frac{1}{2}\vec{u}B  $$

Finally, we simulate reads taken from the *s* samples. For simplicity, we assume the tumor is completely mixed, so that the expected cellular composition of each sample matches that of the tumor. For each sample *i* and for each mutation *j*, we simulate *r*_*ij*_∼*Poisson*(*c*) reads, where *c* is the mean coverage. We then draw the number of variant reads *v*_*ij*_ of mutation *j* in sample *i* from a binomial distribution: *v*_*ij*_∼*Binom*(*r*_*ij*_,*f*_*j*_). The *s*×*n* VAF matrix *F* then contains entries *F*_*ij*_=*v*_*ij*_/*r*_*ij*_.

Additionally, we simulate sampling and sequencing noise by adding *overdispersion* to the binomial distribution. We replace *f*_*j*_ with a beta-distributed random variable with mean *f*_*j*_. The beta distribution parameters *α* and *β* are chosen to be:

$$\begin{array}{*{20}l} \alpha &= \frac{(1-\rho)}{\rho}f_{j} && \beta = \frac{(1-\rho)}{\rho}(1-f_{j}) \end{array} $$

where *ρ*∈(0,1) is the overdispersion parameter. This results in a beta distribution with mean *f*_*j*_ and with variance proportional to *ρ*. We simulate sequencing data with less noise by setting *ρ* closer to 0 and more noise by setting *ρ* closer to 1. The case when *ρ*=0 corresponds to no overdispersion.

### Evaluation of Reconstructed Trees

To quantify the quality of the clonal trees we enumerate, we use the mean ancestor-descendant (A-D) distance [38] between trees in $\mathcal {T}(G_{F})$ and the underlying tree *T*. Note that standard phylogenetic distance measures, including Robinson-Foulds [39], do not apply to clonal trees since they contain internal node labels. To quantify the useful information gained from our solutions, we measure how much more similar trees in $\mathcal {T}(G_{F})$ are to the underlying tree than an equal number of random trees. Formally, with $\overline {AD}(S)$ denoting mean A-D distance between trees in the set *S* and the underlying tree, we define the *A-D improvement* to be

7 $$ \frac{\overline{AD}(\text{random}) - \overline{AD}(\mathcal{T}(G_{F}))}{\overline{AD}(\text{random})}.  $$

A-D improvement measures the proportional decrease in incorrect ancestral relationships relative to the random baseline. For example, an A-D improvement of 0 means that trees in $\mathcal {T}(G_{F})$ are no better than random, while an A-D improvement of 1 means that $\mathcal {T}(G_{F}) = \{T\}$, the correct tree.

## Results

We investigated strict and approximate E-VAFFP solutions both in simulated noisy data and in two real datasets of 3 chronic lymphocytic leukemia (CLL) patients from [29] and 7 clear cell renal cell carcinoma (ccRCC) patients from [30]. We also separately evaluated the usefulness of pruning transitive edges from the strict ancestry graph.

### Evaluation of E-VAFFP Solutions on Simulated Data

We first present findings on the existence and quality of E-VAFFP solutions in simulated noisy DNA sequencing data. We begin by describing how parameters affect the likelihood of finding compatible trees and then address how similar those inferred trees are to the underlying tree. Lastly, we examine how the topology of the underlying tree affects $\mathcal {T}(G_{F})$.

#### E-VAFFP Solution Existence

In simulated data, we found that there are typically no E-VAFFP solutions due to sum condition violations. With more clones, more samples, lower coverage, and higher noise, the probability of finding a solution decreases further. We generated 10000 simulated datasets and ran the ancestry graph method for each parameter value (*n* between 3 and 12, *s* between 1 and 15, coverage between 50× and 200×, and *ρ* between 0 and 0.09). We then computed the proportion of trials with at least one E-VAFFP solution, which we call *solvable* trials. We tested each parameter individually, with default values of *n*=10,*s*=5,60× coverage, and *ρ*=0.

With all parameters at their default settings, the proportion of solvable trials was only 14%. Increasing the coverage caused a dramatic increase in this fraction, up to 47% at 200× coverage. On the other hand, higher overdispersion had a strong negative effect on solvable trials, with 89 of the 10000 trials solvable at *ρ*=0.09. High sample count had an equivalently strong negative impact, with only 103 trials exhibiting an E-VAFFP solution at *s*=15. Corroborating these findings, we also found that E-VAFFP solutions rarely exist in lower-coverage real data, which we discuss in a later section.

#### E-VAFFP Solution Quality

**Fig. 3:**
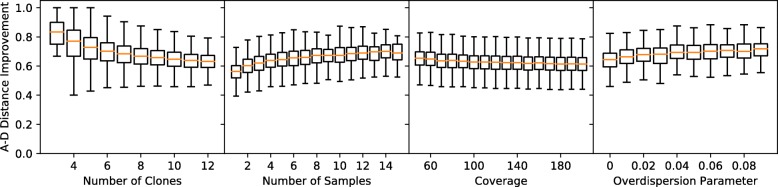
Parameter effects on E-VAFFP solution quality. An A-D improvement of 0 signifies that trees in $\mathcal {T}(G_{F})$ are no better than random, while an improvement close to 1 signifies that $\mathcal {T}(G_{F})$ are nearly identical to the underlying evolutionary tree. Note that solution quality is measured only when solutions exist, which may be rare

We found that when using default parameters, the trees in $\mathcal {T}(G_{F})$ showed a mean A-D improvement of 0.64. This corresponds to a retention of 64% of ancestral patterns in the data missed by the random baseline. Increasing the number of clones *n* makes valid solutions more rare and further decreases the quality of solutions when they do appear. Conversely, increasing the number of samples *s* shows an improvement in the similarity of trees $\mathcal {T}(G_{F})$ to the underlying tree as shown in Fig. 3. The trends we see here for noisy data correspond to those reported by [28] on error-free data.

When we conditioned on the existences of solutions, we counter-intuitively found that higher noise improves solution quality (see Fig. 3). For instance, high coverage slightly decreased A-D improvement, from 0.65 at 50× to 0.61 at 200×. In the rare case that solutions existed, trials with more overdispersion also resulted in better-quality trees, with an A-D improvement of 0.64 at *ρ*=0 and 0.72 at *ρ*=0.09. These findings suggest that spanning trees more similar to the underlying tree are less likely to be rendered invalid by noise. Therefore, noise preferentially disqualifies bad trees from $\mathcal {T}(G_{F})$, resulting in a higher mean A-D improvement. Importantly, the decrease in solution existence is so dramatic that it swamps these modest quality gains, making phylogeny inference worse in high-noise data. For example, the total number of correctly inferred ancestral relationships in all trials does actually decrease as we add more overdispersion, since so few trials are solvable at high *ρ*.

#### Effects of Underlying Tree Topology on E-VAFFP Solutions

**Fig. 4:**
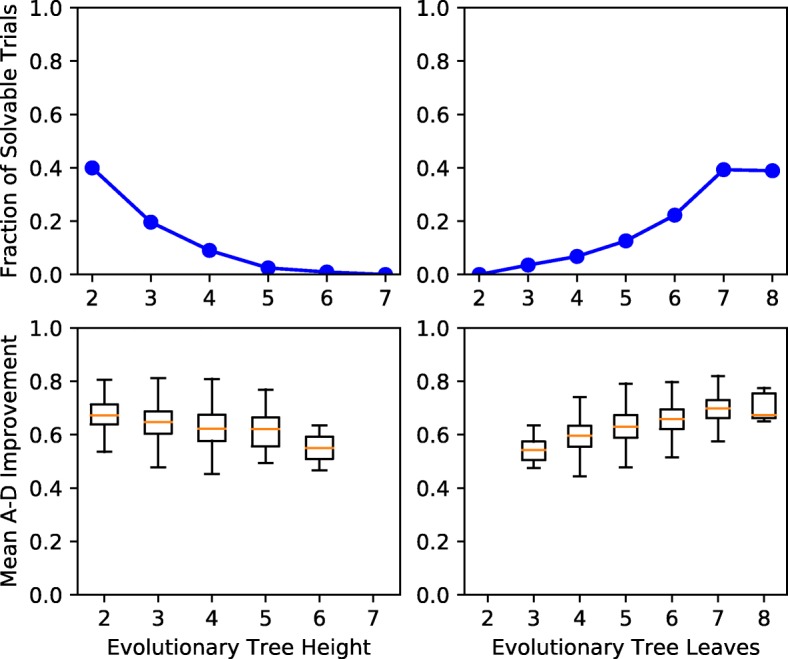
Effects of tree topology on E-VAFFP solution existence and quality. The top row shows the effects of underlying tree height and leaf count on the fraction of trials with any compatible trees. The bottom row shows the effects of these tree metrics on solution quality. Shallow, wide trees yield better reconstructions

The topology of a tumor’s underlying evolutionary tree can have a strong effect on the accuracy of reconstruction methods. Trees that are wider (more leaves) and shallower (lower tree height) than average randomly generated trees are said to have a *branching* topology. We find that the features of a branching topology are more likely to result in E-VAFFP solutions and have improved solution quality (see Fig. 4). We also performed these analyses using two additional measures of tree topology, single child fraction and mean subtree height. Similarly, we found that trees that have a low single child fraction and a low mean subtree height (both features of branching trees) also are more likely to yield E-VAFFP solutions and have improved solution quality (see Additional file 1).

The reason why E-VAFFP solutions perform better on branching trees is not immediately obvious. One possible explanation relates to the effect of simulated noise on the resulting ancestry graph. If a descendant mutation and its ancestor have very similar mutation frequencies, then even a small amount of noise could reverse the order of the observed frequencies, violating the ancestry condition. In a totally linear tree, it is possible that each pair of connected vertices has similar frequencies in some sample. Thus, every edge in the ancestry graph has the potential to be reversed by noise. In contrast, in a totally branching tree, since all children vertices must adhere to the sum condition, only one child can have a frequency very similar to its parent. Thus, fewer edges in the ancestry graph are likely to be prone to noise. Hence, trees that exhibit more branching may appear more robust under the E-VAFFP than linear trees.

### Evaluation of Approximate Solutions on Simulated Data

Just as with strict E-VAFFP solutions, we examined the quality of solutions derived from the approximate ancestry graph. We also determined the viability of relaxing the sum condition and investigated the validity of the edge weighting function used to construct the approximate graph.

#### Approximate Solution Existence

Even when the error threshold *ε* is small, the relaxed sum condition (5) results in a significant increase in the proportion of solvable trials. We let *ε* range from 0 and 0.05, since real data indicated that sum condition overflows are typically small in practice (Table 2). As we increased *ε* in this range, we observed a proportional increase in the fraction of solvable trials from 14% to 64%. However, there was also a significant increase in the mean number of trees in $\mathcal {T}(G_{F})$ from 2000 to 69000, which dramatically slows down inference. Thus, there is a trade off between the probability of finding a valid tree and computational cost of enumerating these trees.

#### Approximate Solution Quality

**Fig. 5:**
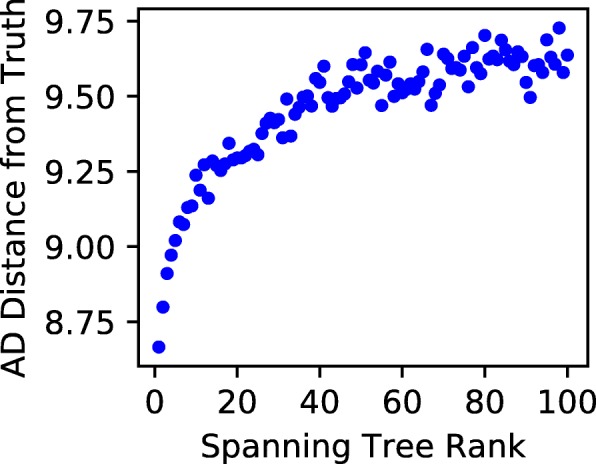
Relationship between approximate ancestry graph tree rank and solution tree quality. High-weight trees are more similar to the underlying tree than low-weight trees, although the trend levels off rapidly

The approximate ancestry graph method is founded on the assumption that the weighting function in Eq. 4 accurately represents the probability that the corresponding edge exists in the underlying evolutionary tree. If this is the case, the total weight of a solution tree should be a measure of its quality, and the max-weight tree should be the most similar to the underlying tree. We verified this by comparing the ranks of approximate ancestry graph spanning trees to their the mean A-D distance from the underlying tree. We say that the max-weight valid spanning tree has rank 1 and that the *i*th highest weight valid tree has rank *i*. We selected the 1104/10000 trials with at least 100 valid spanning trees and sorted the top 100 trees in descending weight order. Then, we aggregated statistics for each tree rank across trials. As expected, we found that high-weight trees are in fact more similar to the underlying tree than lower weight trees (Fig. 5). Specifically, we find that the average rank 1 tree has 6.9% smaller A-D distance to the underlying tree than the average rank 25 tree. This effect begins to level off as rank increases: the average rank 25 tree has just 3.4% smaller A-D distance to the underlying tree than the average rank 100 tree.

**Fig. 6:**
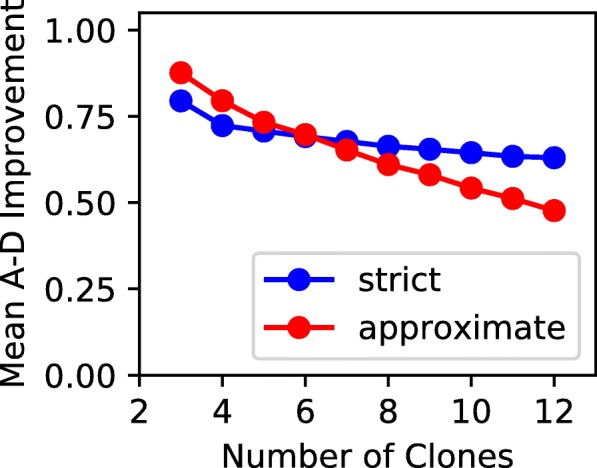
Difference in relationship between *n* and A-D improvement with strict and approximate ancestry graph methods. As the number of clones increases, both methods worsen, but the approximate ancestry graph does so more rapidly

We also examined the effects of parameters on the quality of approximate solutions. Solution quality responds in the same way to changes in sample count, coverage, and overdispersion in the approximate ancestry graph as in the strict ancestry graph. However, we found an intriguing difference in the response to number of clones *n*. Choosing the max-weight valid spanning tree of the approximate graph provides noticeably better solutions than the strict approach for small *n*. However, the approximate method drops off more sharply in quality as *n* grows, with the crossover point at *n*=6 (see Fig. 6). We suspect this is due to inherent bias in high-weight approximate spanning trees, since they become worse than randomly sampled strict spanning trees (as measured by A-D improvement) as *n* grows. We investigate this phenomenon in depth in the following section. We also found that relaxing the sum condition caused a gradual linear decrease in the approximate solution quality, from an A-D improvement of 0.54 at *ε*=0 to 0.51 at *ε*=0.05 when the number of clones is *n*=10. The negative effect on quality of relaxing the sum condition lessens when there are fewer clones, and the relaxed sum condition may even improve mean solution quality when *n*<6 (see Additional file 3).

#### Tree Rank in the Approximate Ancestry Graph

As we saw in Fig. 6, the quality of solutions derived from the approximate ancestry graph falls off more quickly than the strict E-VAFFP enumeration method as the number of clones increases. We believe this is due to systematic bias in high-weight spanning trees. This bias may arise because edges in the approximate graph are weighted by the probability that one clone is ancestral to another, but that edges in fact represent parental rather than ancestral relationships. As such, the root node is likely to have high-weight edges to every other node, even though its probability of being their direct parent may not be as high. This would result in high-weight spanning trees that tend to be shallow and wide.

**Fig. 7:**
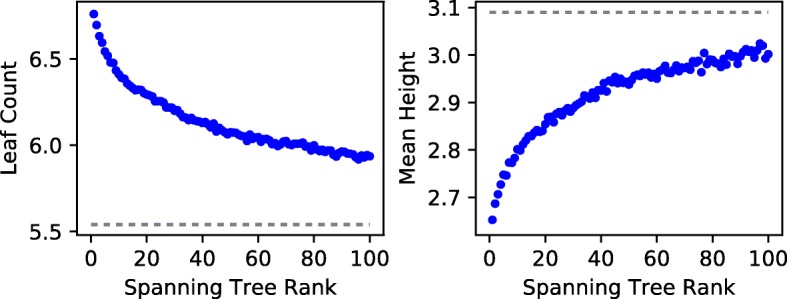
Relationships between approximate ancestry graph tree rank and solution tree topology. The dashed lines show the average values for underlying trees yielding at least 100 spanning trees. On average, high-weight solutions are wider and shallower than lower height solutions. Additionally, they are significantly wider and shallower than the underlying evolutionary trees

To assess this conjecture, we gathered data on the relationship between spanning tree rank in the approximate graph and the four topology metrics from the previous section (height, leaf count, single child fraction, and mean subtree height). We found a strong and consistent trend that high-weight trees do in fact tend to be shallower and wider than lower weight trees (Fig. 7). This effect is most pronounced at low ranks, with average heights of 2.65 at rank 1, 2.88 at rank 25, and 3.00 at rank 100. Moreover, the average underlying tree yielding at least 100 solutions has height 3.09. This shows that high-weight spanning trees are biased towards branching topologies. We found the same trend using leaf count, mean subtree height, and single child fraction. Despite this bias, the max-weight tree is still, on average, the best choice available among approximate ancestry graph spanning trees, as seen in the previous section.

### Evaluation of Transitive Edge Pruning

**Fig. 8:**
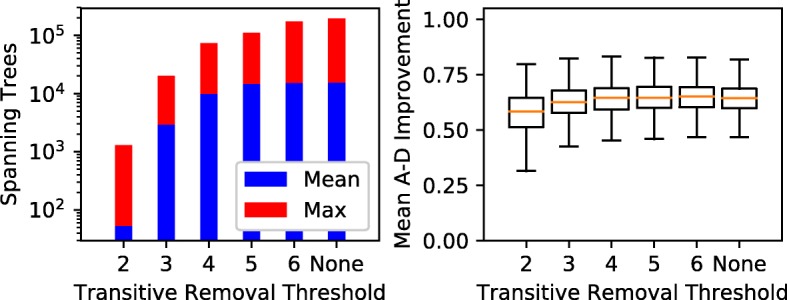
Effect of partial transitive reduction on the number and quality of solutions. ‘None’ represents the unpruned ancestry graph

We found that partial transitive reduction (PTR) successfully reduces the size of $\mathcal T (G_{F})$ while preserving solution quality. We first compared the solution quality and existence that result from applying PTRs to the standard ancestry graph method. Next, we counted the average and maximum number of ancestry graph spanning trees as a measure of performance improvement due to PTR (Fig. 8). Our default parameters were unchanged from the previous experiment.

The 2-PTR (i.e. the canonical transitive reduction) was too extreme to be useful, as it decreased the fraction of solvable trials to 3%. Moreover, 2-PTR also decreased solution quality as measured by mean A-D improvement from 0.64 in the standard ancestry graph to 0.57 (Fig. 8).

On the other hand, higher-order PTR (6+) had almost no effect, as ≥6-transitive edge are exceedingly rare in ancestry graphs with 10 nodes. However, 4- and 5-PTR showed more promise. Neither had a noticeable impact on the proportion of solvable trials, but they reduced the maximum spanning tree count by 43% and 62%, respectively. At the same time, both 4- and 5-PTR decreased the mean A-D improvement by less than 0.01. The 3-PTR had a correspondingly stronger impact on these quantities, decreasing the mean and maximum spanning tree counts by factors of 7.7 and 9.6 relative to the standard ancestry graph. The proportion of solvable trials shrank by two percentage points with 3-PTR, while the mean A-D improvement was 0.02 worse.

To summarize, we were able to reduce the number of edges in 10-node ancestry graphs without harming solution quality and existence using 3-, 4-, and 5-PTR. Fewer edges results in fewer spanning trees, and thus lower runtime, less memory usage, and the potential to handle more clones. Picking different partial transitive reductions allows us to control the trade-off between these benefits and better solutions. With a different number of nodes, we would have to pick a different PTR to achieve the desired balance.

#### Topology Effects of PTR

**Fig. 9:**
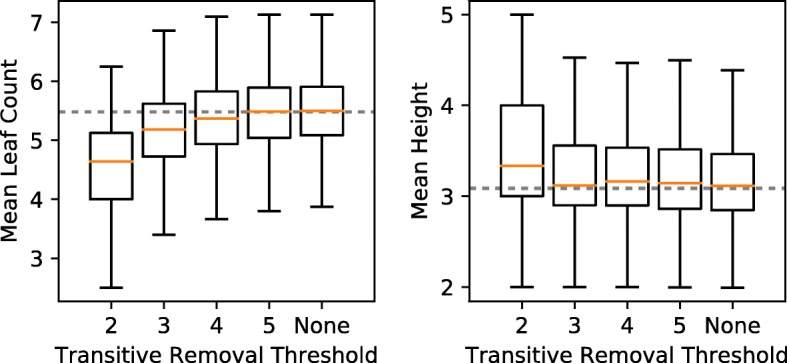
Effect of partial transitive reduction on topological features of $\mathcal {T}(G_{F})$. PTR disproportionately removes wide trees, causing the mean leaf count to decrease with more extreme pruning. The effect on tree height is less clear, although 2-PTR clearly removes shallow trees. The dashed line shows the mean value for underlying trees

Removing highly transitive edges from the ancestry graph disproportionately removes wide spanning trees from $\mathcal {T}(G_{F})$ (Fig. 9). We considered 2-, 3-, 4-, and 5-PTR across 10000 trials of 10-node ancestry graphs. In particular, we only report results across trials in which solutions existed after pruning transitive edges (267, 1183, 1360, and 1409/10000 for 2-, 3-, 4-, and 5-PTR, respectively). We found that 2-PTR (the most extreme reduction) results in valid trees with 0.80 fewer leaves on average, while 3-PTR reduces the mean number of leaves by 0.33. In contrast, the mean height of solution trees only seems to be significantly affected by 2-PTR, which increased the mean height of trees by 0.25. For 3- and higher-order PTR, the mean height of trees was affected by less than 0.06. Single child fraction and mean subtree height both display similar trends to leaf count (see Additional file 1).

### Real Data

We evaluated the strict and approximate ancestry graph methods using a chronic lymphocytic leukemia (CLL) dataset [29] and a clear cell renal cell carcinoma (ccRCC) dataset [30]. For the CLL data, we examined VAFs from 100000× coverage targeted deep sequencing and from 40× coverage whole genome sequencing (WGS). The ccRCC dataset used amplicon sequencing, with over 400× average coverage [30]. An overview of the two datasets can be found in Table 1. For both datasets, we used the approximate and strict ancestry graph approaches to enumerate candidate clonal trees. When the standard sum condition yielded no solutions, we instead applied the relaxed sum condition (5), picking the smallest *ε* that resulted in at least one ancestry graph spanning tree. In the CLL data, we clustered mutations by observed frequency across all samples using *k*-means, and manually chose the number of clusters. For the ccRCC dataset, we instead used the clusters found by LICHeE, which uses mutation occurrence to enhance VAF-based clustering [19]. We note that we could have chosen to use a different method for mutation clustering (e.g. PyClone [40]) for this analysis. However, we choose the clusters produced by LICHeE as this allowed a direct comparison of our reconstructed trees with those reported in the LICHeE paper, which also analyzed this dataset. Furthermore, we note that PyClone is designed for more deeply sequenced mutations than we had available here. For both datasets, these clusters represent hypothesized clones in the tumor. To remove sites that may have undergone copy number aberrations, we ignored all mutations with a VAF above 0.5.

**Table 1:** Dataset Summary

Patient	Samples	Mutations	Clones	$|\mathcal {T}(G_{F})|$
CLL003 (D)	5	15/20	4	0
CLL003 (W)	5	13/30	4	0
CLL006 (D)	5	5/10	5	2
CLL006 (W)	5	6/16	5	0
CLL077 (D)	5	12/16	4	1
CLL077 (W)	5	16/20	4	0
EV003	8	33/49	8	0
EV005	7	58/75	8	0
EV006	9	57/72	7	0
EV007	8	48/60	10	0
RK26	11	58/62	12	0
RMH002	5	44/51	8	0
RMH008	8	69/77	10	0

Mutation counts are displayed after/before filtering out mutations with VAF above 0.5. Mutations in CLL patients were clustered by VAF using *k*-means to identify clones, while we used clusters from [19] for the ccRCC patients. (D) indicates deep sequencing data and (W) indicates WGS data

**Table 2:** ccRCC tree comparison with LICHeE

Patient	*ε*	Notes
EV003	0.037	Exact match.
EV005	0.046	One node different.
EV006	0.078	Exact match.
EV007	0.042	Exact match.
RK26	0.028	One node different.
RMH002	0.086	Exact match.
RMH008	0.027	Exact match.

The second column shows the sum condition relaxation required. The third column notes the degree of similarity between our inferred tree and that of LICHeE

#### Rarity of Strict Solutions

Of the 11 patients we analyzed, only the 100000× coverage targeted sequencing data for CLL006 and CLL077 admitted E-VAFFP solutions. In all other cases, we had to use the approximate ancestry graph and relax the sum condition in order to find likely clonal trees. This pattern agrees with the finding in simulated data that E-VAFFP solutions are rare and reinforces the importance of coverage in solution existence.

For the datasets in which an E-VAFFP solution existed, we observed one compatible tree in the CLL077 data (with four clones) and two trees in the CLL006 data (with five clones). For comparison, in simulated data, 19% of the *n*=4 solvable trials had one tree and 12% of the *n*=5 solvable trials had two trees.

#### WGS and Targeted Sequencing Agreement in CLL Data

**Fig. 10:**
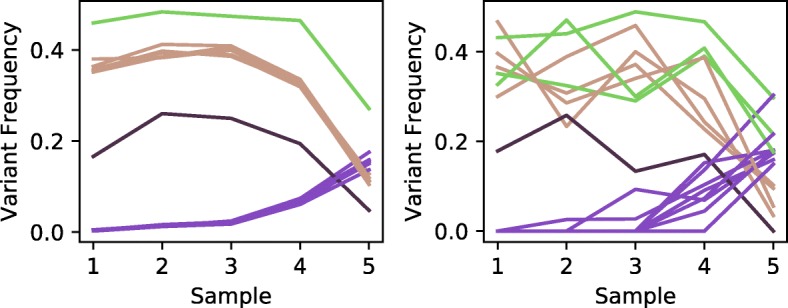
Variant allele frequencies over five samples for patient CLL077. The left panel shows VAFs from targeted deep sequencing and the right panel shows VAFs from whole genome sequencing [29]. The colors of arcs indicate which mutations were clustered together using *k*-means

**Fig. 11:**
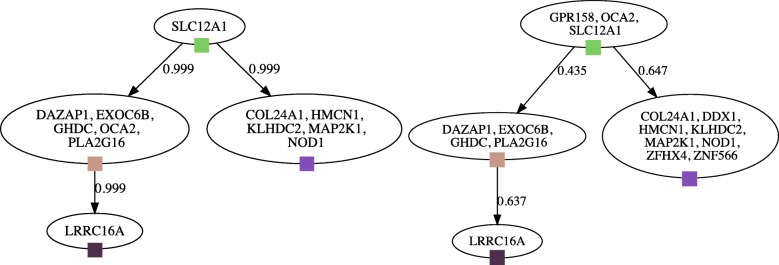
Clonal trees identified for patient CLL077. The left panel shows the tree derived from deep sequencing and the right panel shows the tree from WGS data. These trees were the max-weight spanning trees of the respective approximate ancestry graphs. Edge weights are the probability of the relationship and color labels correspond to clusters in Fig. 10. The movement of OCA2 to the root is due to different clustering as a result of noise (see Fig. 10). DDX1, ZFHX4, and ZNF566 were not represented in the deep sequencing data, while GPR158 was filtered out in the deep sequencing data due to VAF over 0.5. The WGS tree required a sum condition relaxation of *ε*=0.048

The trees identified from both WGS and deep sequencing data for all three CLL patients were toplogically identical, regardless of whether we had inferred them using the strict or approximate methods. All minor labeling differences were the result of mutations that were filtered or simply absent in one of the datasets or that were differently clustered because of noise in the WGS data. Figure 10 displays the variant frequencies in patient CLL077, which showcases high WGS data noise. See Fig. 11 for the trees inferred from the deep and WGS CLL077 data.

Furthermore, setting aside any mutations filtered out because of possible copy number aberrations, the CLL trees we found agree entirely with the trees identified by two other inference methods, CITUP [18] and PhyloSub [20]. Moreover, our CLL077 tree displays the two major branches inferred by AncesTree [17]. Most significantly, our CLL003 tree, which we generated with the approximate ancestry graph and the relaxed sum condition, precisely matches the trees found by PhyloSub and CITUP.

It is worth emphasizing that when we relaxed the sum condition in the CLL006 and CLL077 WGS data, we recovered the same trees that had obeyed the sum condition in the deep sequencing data. Noise in the WGS data introduced sum condition violations of 0.101 and 0.048 in the CLL006 and CLL077 data, respectively. This is evidence that our sum condition relaxation, in concert with the approximate ancestry graph, allows us to successfully infer likely trees despite noise rendering the sum condition unsatisfiable. It is also worth mentioning that the CLL trees had few clusters, only 4 or 5. This places us within the regime we found in simulated data where the approximate method performs better than the strict method (see Fig. 6).

### Approximate Solutions in ccRCC Data

As noted earlier, none of the eight ccRCC patients’ data admitted strict E-VAFFP solutions. However, relaxing the sum condition and using the approximate ancestry graph nonetheless allows us to find candidate clonal trees. We selected the smallest sum condition relaxation *ε* that resulted in a single valid tree. In the case that several trees were found with the same sum condition relaxation, we picked the one with the highest weight in the approximate ancestry graph.

The trees we found in this way for patients EV003, EV005, EV006, EV007, RMH002, RMH008, and RK26 display strong agreement with those found by LICHeE [19]. See Table 2 for the tolerance *ε* needed to find these trees and for notes on their agreement with LICHeE (the trees themselves can be found in Additional file 2). We did not compare our results on RMH004 to those reported by LICHeE due to an apparently malformed data file used to create those results. The sum condition overflows in the ccRCC data were relatively small (the largest *ε* required was 0.086) but consistently present across patients. However, it is difficult to determine whether these overflows are due to legitimate ISA violations, such as the occurrence of convergent mutations, or simply due to noise in the measured VAFs.

## Discussion

In simulated data, we confirmed that high noise decreases the probability of strict clonal tree existence. However, in the rare case that trees can be identified in high-noise data, they tend to be better than the more common trees found from low-noise data. This shows that trees similar to the underlying tree are more robust to noise than dissimilar trees. Additionally, we found that the topology of the underlying tree has a strong impact on the quality and ease of phylogeny inference. While our analysis here focuses on the ancestry graph approach introduced in [17], the sum condition that underlies that method (which results from the ISA) is shared by a number of other approaches, such as [18–20] and others. Therefore, our conclusions here may likely apply to other methods—including new phylogenetic inference methods continuing to be developed. Thus, we claim that patterns of tumor evolution (linear, branching, etc.) should be more explicitly considered when developing and applying inference methods. This may become increasingly important as large-scale studies look across patients to identify common patterns of evolution within and across cancer types.

Meanwhile, we showed that the approximate ancestry graph method provides better trees than the strict approach when there are few clones and worse trees when there are many clones. This is likely connected to the relationship we found between tree rank and topology, with high-weight trees likely to be wide and shallow. Moreover, the approximate graph produces trees significantly skewed in this direction.

We also found several results that bear on the validity and applicability of the ISA. Despite the fact that our simulated data procedure adhered to the ISA, the majority of resulting VAF data broke the sum condition due to the noise added to the simulation. We found the same kind of violations in the ccRCC and 400× coverage CLL data (with the notable exception of the ultra-high 100000× coverage CLL data). However, we still found clonal trees in agreement with existing literature using only small sum condition overflows *ε*, no higher than 0.09. This indicates that some violations of strict frequency assumptions are to be expected even if the ISA largely holds in practice. These findings encourage the exploration of methods that relax the ISA, although it is not clear that we should abandon it entirely.

We hope that our analysis here will be useful to those analyzing and interpreting real tumor phylogenies constructed using methods that rely on the ISA. Several unanswered questions remain. For instance, we observed that higher coverage decreased the average number of correctly reported ancestral relationships. We are curious to know if this trend continues with more extreme coverages and to understand why this occurs. Future work should also address the impact of noise, tree topology, and other parameters on methods that relax the ISA or that consider mutations more complex than SNVs, such as copy number aberrations. Furthermore, our data simulation procedure did not include complex effects such as regional tumor heterogeneity or distinguish between driver and passenger mutations. These other factors could effect phylogeny inference and merit additional investigation. Finally, while we focused on methods applicable to multi-sample bulk sequencing data, the analysis of these issues with regard to long-read and single-cell sequencing data will need further attention as these technologies become increasingly feasible, since both show promise in improving phylogeny inference [28].

## Conclusions

We explored the inference of tumor evolutionary history from SNV frequency data obtained from multi-sample bulk sequencing using the ancestry graph method of [17]. This method is founded on the infinite sites assumption (ISA) and further simplifies the problem by ignoring copy number aberrations. Our contributions here include introduction and exploration of two methods of loosening the strict ISA assumption that allowed phylogenies to be found even in non-idealized data. We evaluated the effects of parameters, noise, and evolutionary tree topology on the existence and quality of candidate clonal trees. We found that these factors can significantly influence phylogeny inference, often in non-obvious ways (e.g. the counterintuitive effects of high coverage and high noise on solution quality). Methodically, we defined the partial transitive reduction of a graph and showed that it can be used to simplify the ancestry graph while on average preserving spanning trees similar to the underlying evolutionary tree. We applied these methods to real cancer datasets, confirming our findings in simulated data about the existence of strict solutions and the viability of the approximate approach.

Tumor phylogeny inference has the potential to yield insight into how tumors develop and potentially to inform personalized cancer treatment [8, 9], which will become increasingly viable as sequencing methods continue to improve and become cheaper. As such, it is important not only to develop new and more accurate inference methods, but also to understand how those methods are impacted by the data they take as input. However, this issue has not been thoroughly explored in the existing literature [28]. Our work here addresses this oversight explicitly and has numerous potential implications. Our findings on the effects of controllable factors like sequencing coverage and number of sequenced samples can help inform practical decisions in real-world phylogeny inference experiments. For instance, we found that higher coverage does not necessarily improve the quality of inferred trees. Additionally, our results on uncontrollable factors like tumor evolution patterns and clone count can assist in interpreting trees reconstructed using ISA-based approaches such as [17–20]. Finally, our results provide strong motivation for additional work in exploring the performance of inference methods under different situations, since we showed that factors like tumor evolution pattern and noise levels exert significant pressure on inference results.

## Supplementary information

**Additional file 1** Single child fraction and mean subtree height plots. This PDF file contains corresponding plots for each of our topology results using these additional measures of tree topology.

**Additional file 2** CLL and ccRCC trees. This PDF file contains the trees we reconstructed from real data, as well as the trees we obtained by running LICHeE.

**Additional file 3** Joint effect of clone count and sum condition relaxation. This PDF file contains plots showing the interaction effects of the clone count *n* and the sum condition relaxation parameter *ε*.

## Data Availability

The simulated data and the SNV read counts from the real datasets supporting the conclusions of this article are available here: https://bitbucket.org/oesperlab/inference-effects-data.

## Abbreviations

(E-)VAFFP: (Enumeration) variant allele frequency factorization problem
A-D: ancestor-descendant
ccRCC: Clear cell renal cell carcinoma
CLL: Chronic lymphocytic leukemia
DAG: Directed acyclic graph
ISA: Infinite sites assumption
PTR: Partial transitive reduction
SNV: Single nucleotide variant
VAF: Variant allele frequency
WGS: Whole genome sequencing
