# Supplementary material for: Parameter, noise, and tree topology effects in tumor phylogeny inference
Source: BMC Med Genomics. 2019 Dec 23;12(Suppl 10):184. doi: 10.1186/s12920-019-0626-0 (PMC6927103; doi:10.1186/s12920-019-0626-0)
Supplement: Supplementary file 1 — Additional file 1 Single child fraction and mean subtree height plots. This PDF file contains corresponding plots for each of our topology results using these additional measures of tree topology. [file 12920_2019_626_MOESM1_ESM.pdf]

Single Child Fraction and Mean Subtree Height Plots

The paper gives tree topology results in terms of height and leaf count. This file contains corresponding plots for two other measures of tree topology, single child fraction and mean subtree height. The *single child fraction* of a tree is the proportion of nodes with exactly one child. The *mean subtree height* is the average over all nodes of the height of the subtree rooted at that node. Trees with branching topologies have low single child fraction and low mean subtree height.

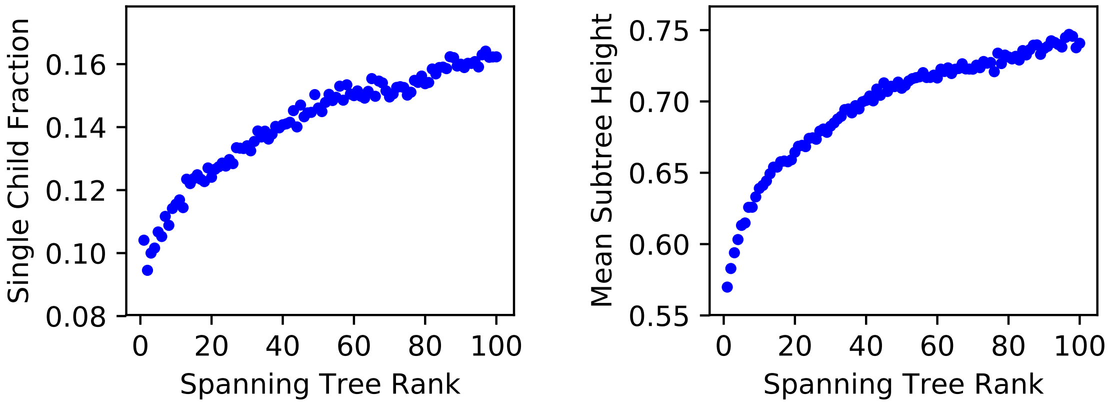

Figure 1: Tree rank in the approximate ancestry graph.

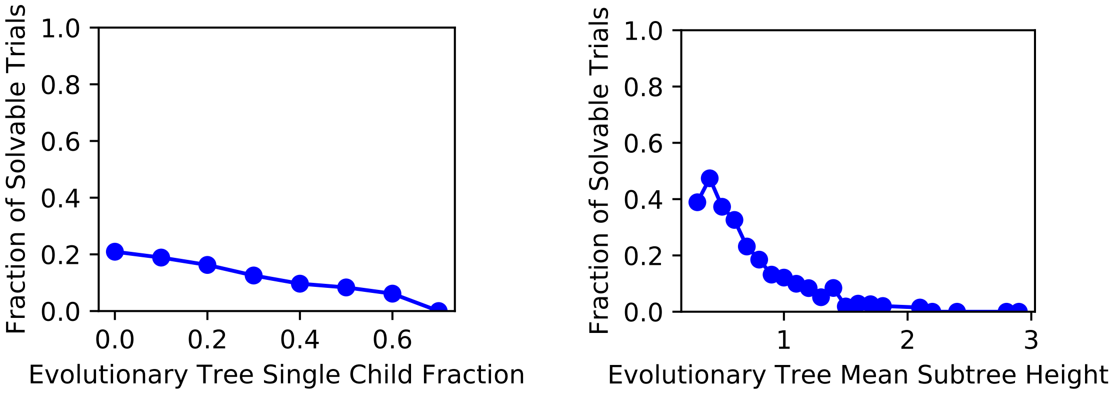

Figure 2: Effect of tree topology on E-VAFFP solution existence.

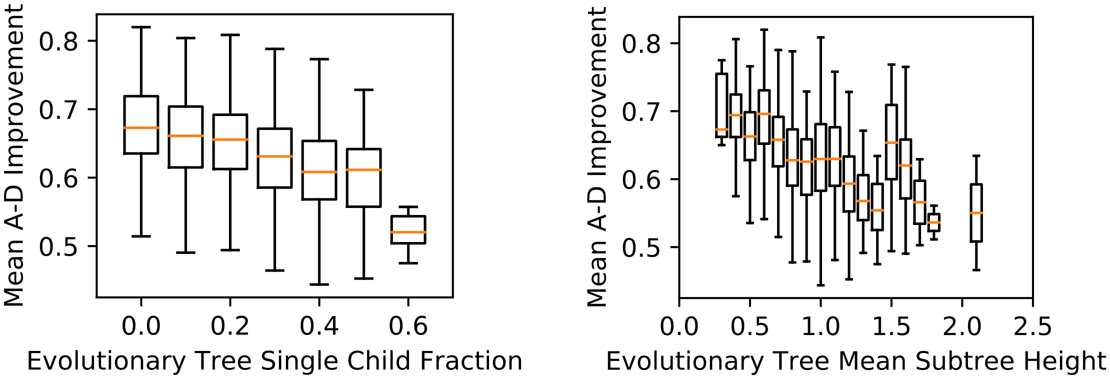

Figure 3: Effect of tree topology on E-VAFFP solution quality.

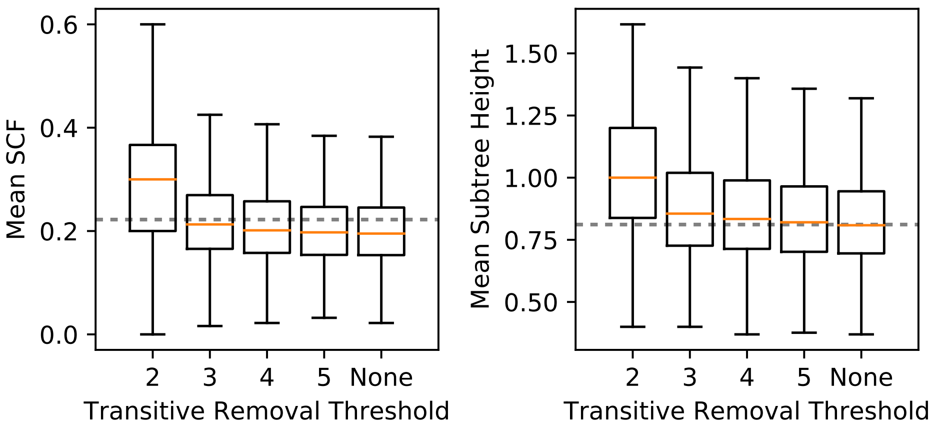

Figure 4: Effect of partial transitive reduction on solution tree topology.
