# Supplementary material for: Parameter, noise, and tree topology effects in tumor phylogeny inference
Source: BMC Med Genomics. 2019 Dec 23;12(Suppl 10):184. doi: 10.1186/s12920-019-0626-0 (PMC6927103; doi:10.1186/s12920-019-0626-0)
Supplement: Supplementary file 2 — Additional file 2 CLL and ccRCC trees. This PDF file contains the trees we reconstructed from real data, as well as the trees we obtained by running LICHeE. [file 12920_2019_626_MOESM2_ESM.pdf]

CLL and ccRCC Trees

1 CLL Trees

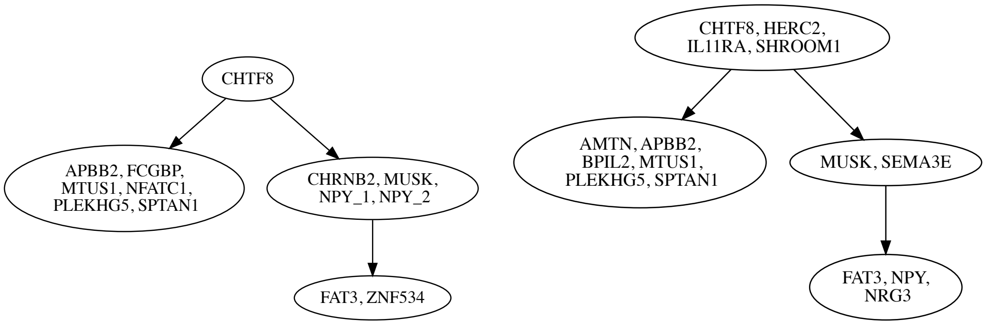

(a) WGS (b) Deep

Figure 1: CLL003

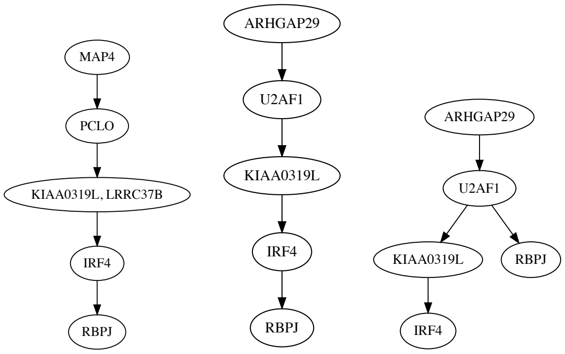

(a) WGS (b) Deep Tree 1 (c) Deep Tree 2

Figure 2: CLL006

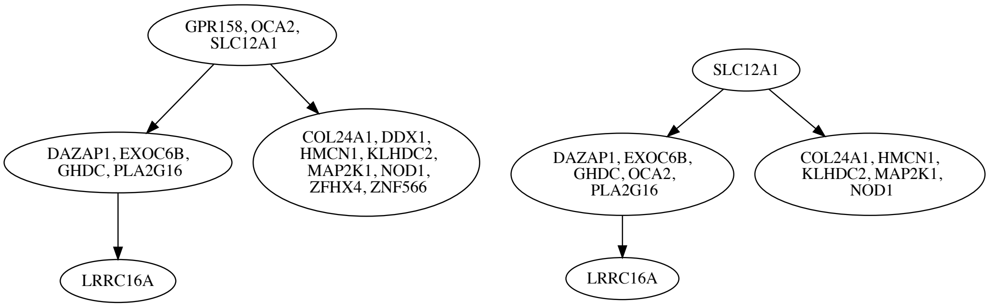

(a) WGS (b) Deep

Figure 3: CLL077

2 ccRCC Trees

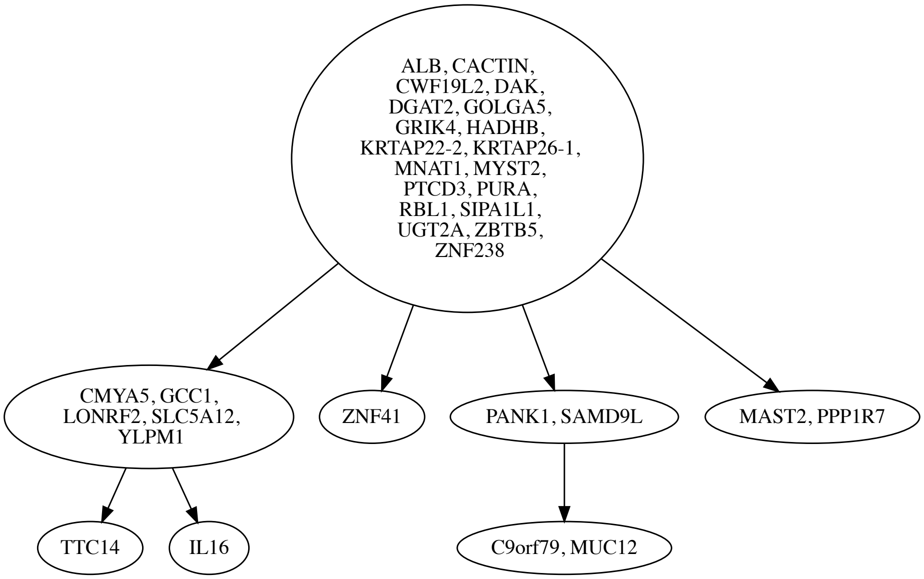

(a) Ours

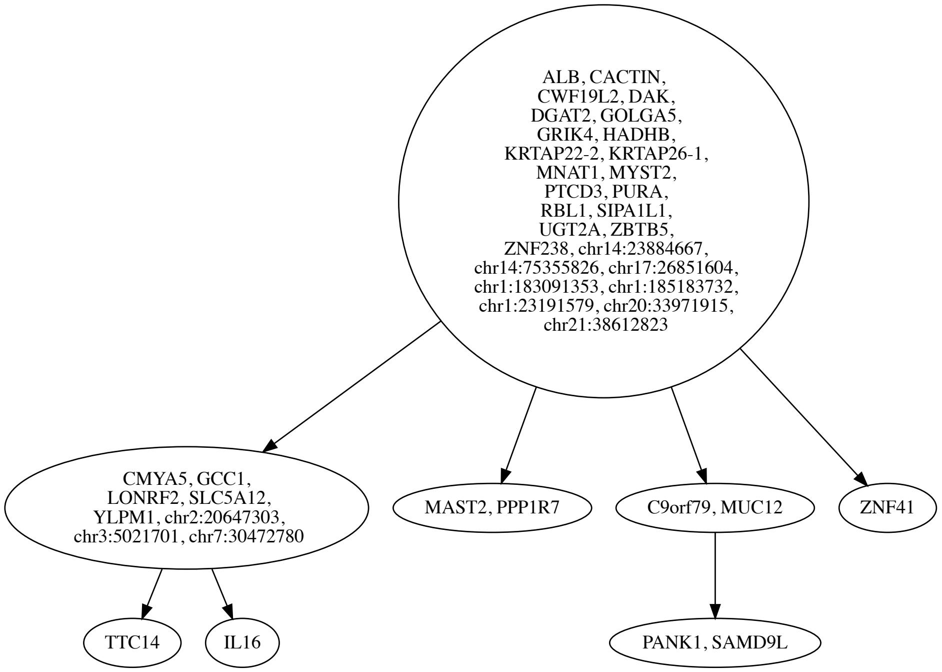

(b) LICHeE

Figure 4: EV003

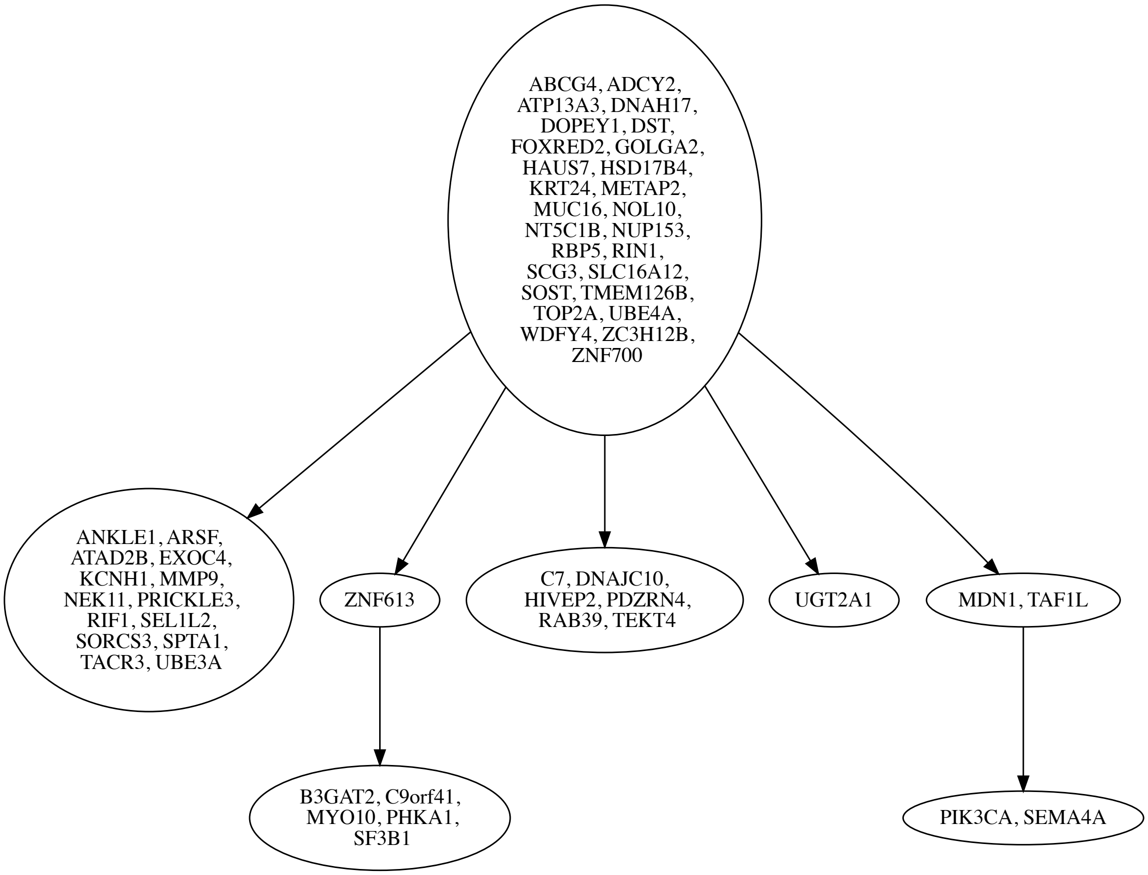

(a) Ours

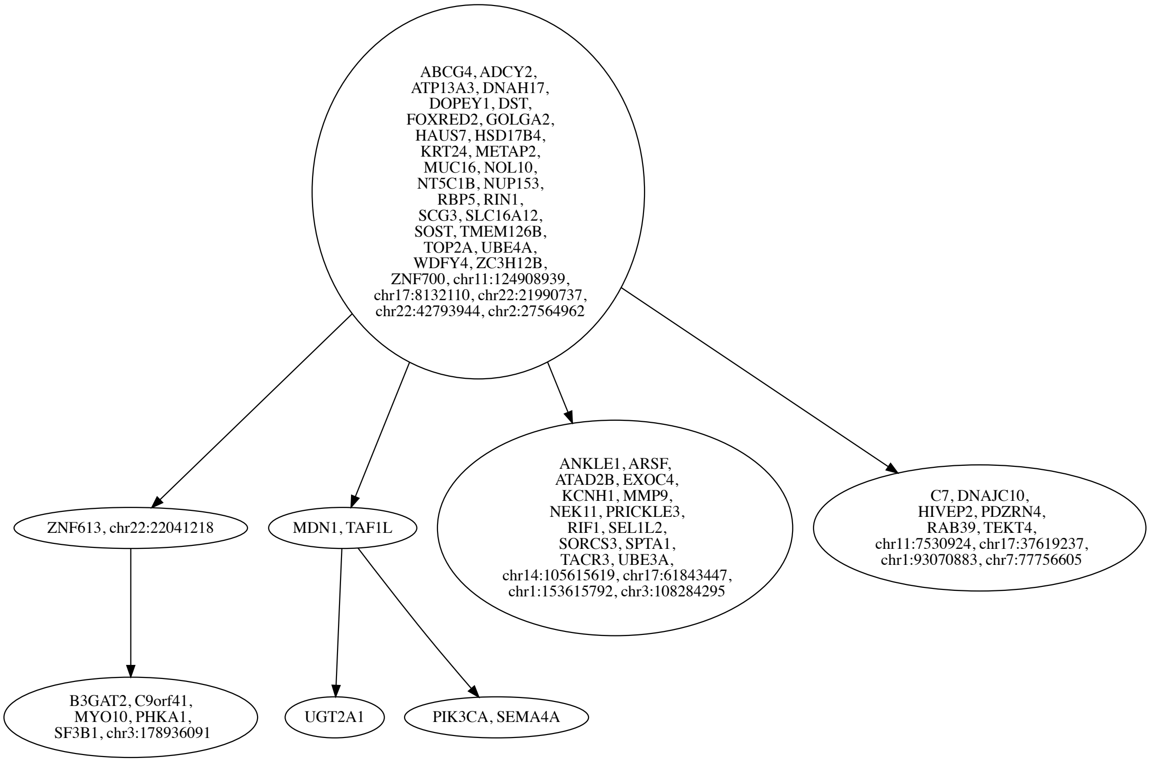

(b) LICHeE

Figure 5: EV005

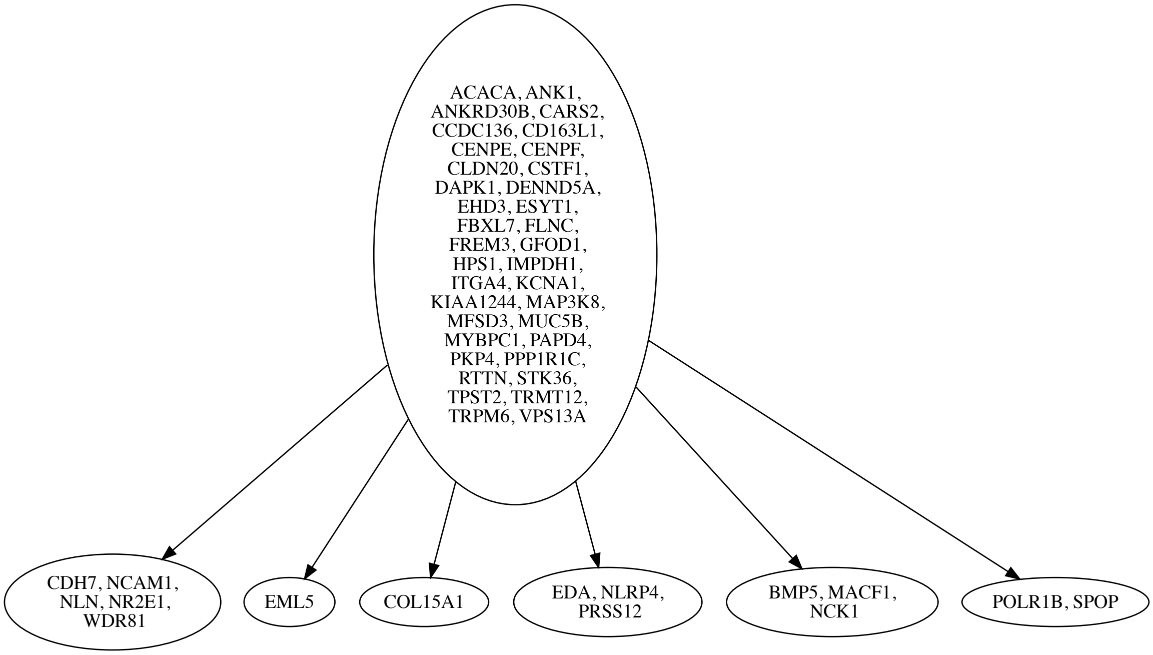

(a) Ours

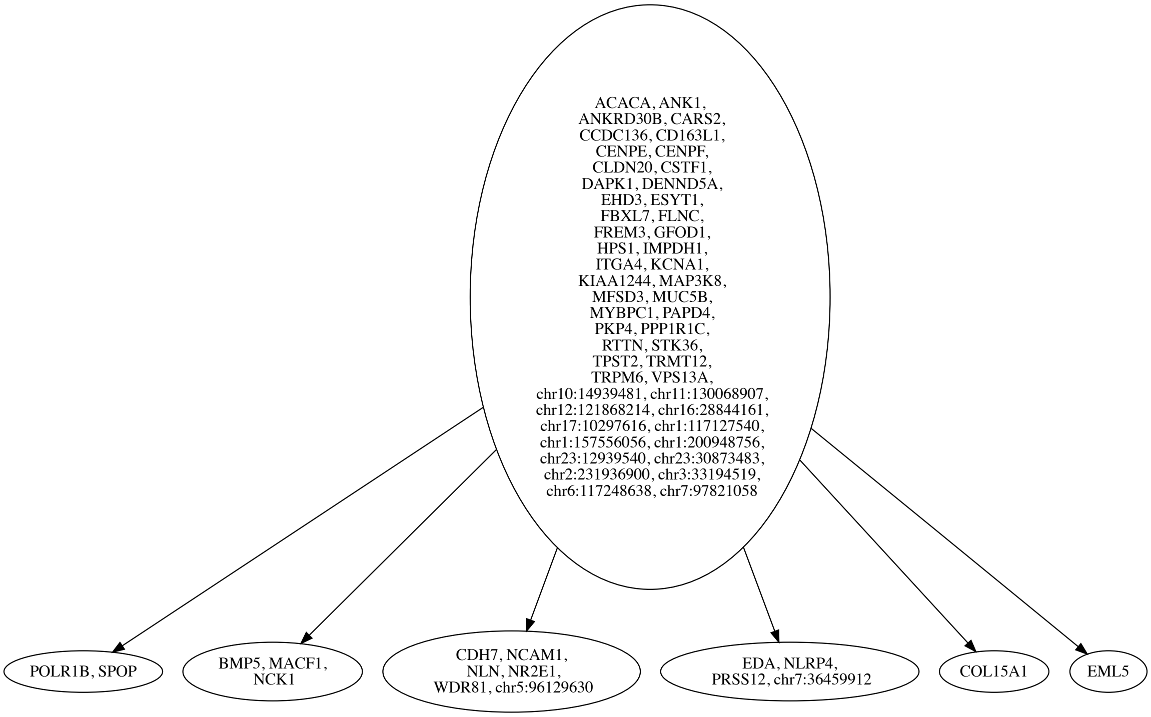

(b) LICHeE

Figure 6: EV006

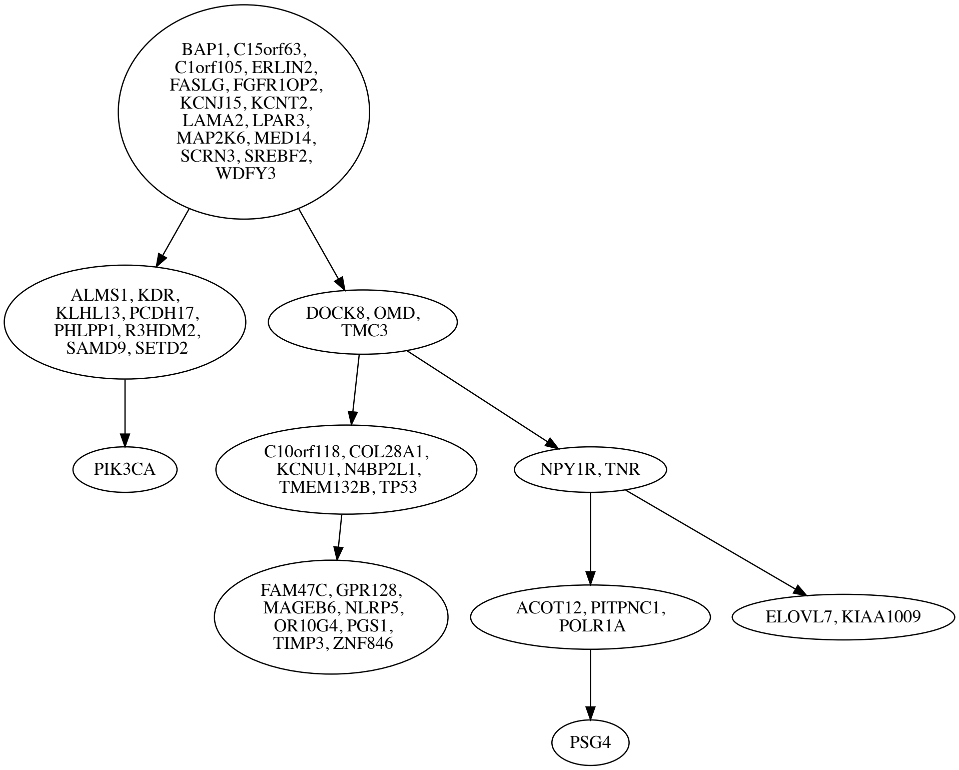

(a) Ours

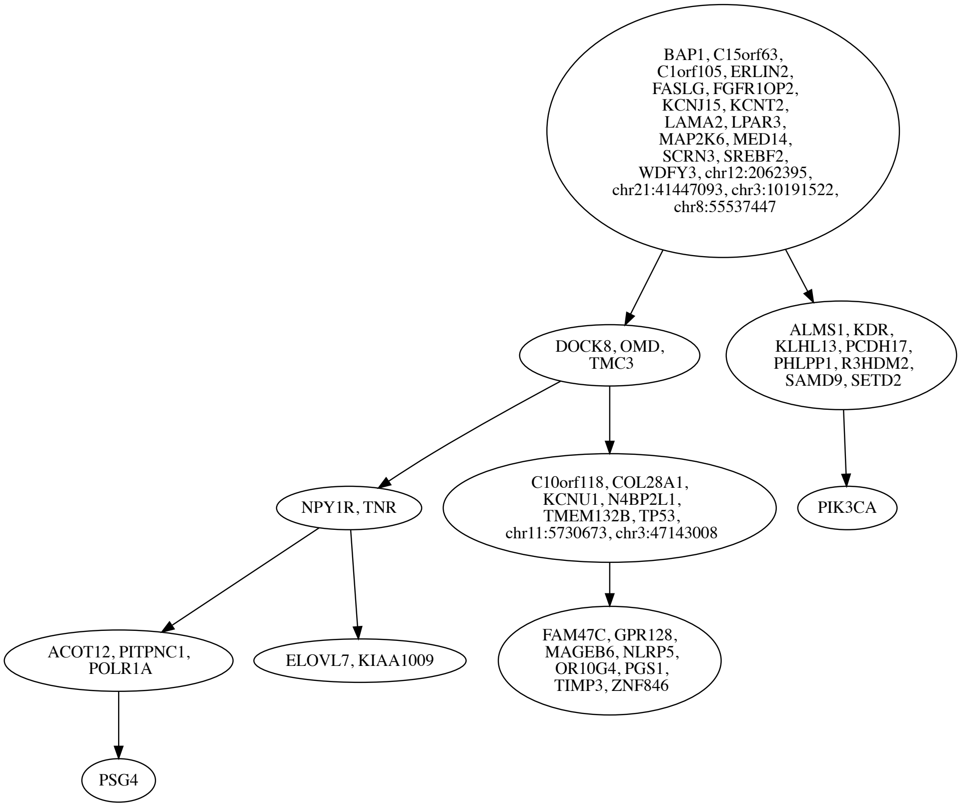

(b) LICHeE

Figure 7: EV007

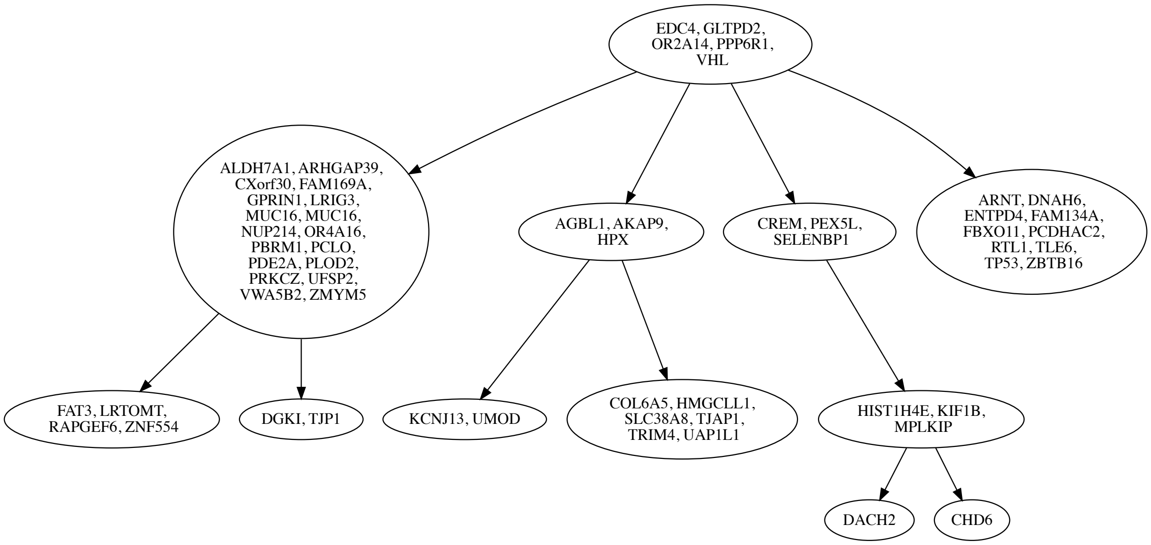

(a) Ours

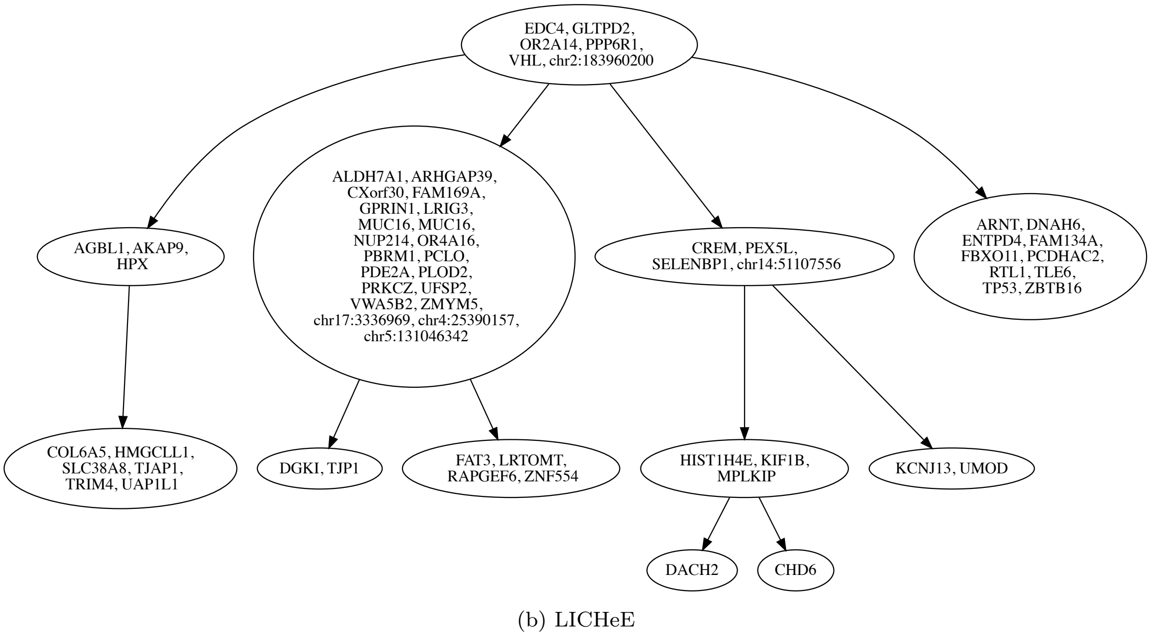

(b) LICHeE

Figure 8: RK26

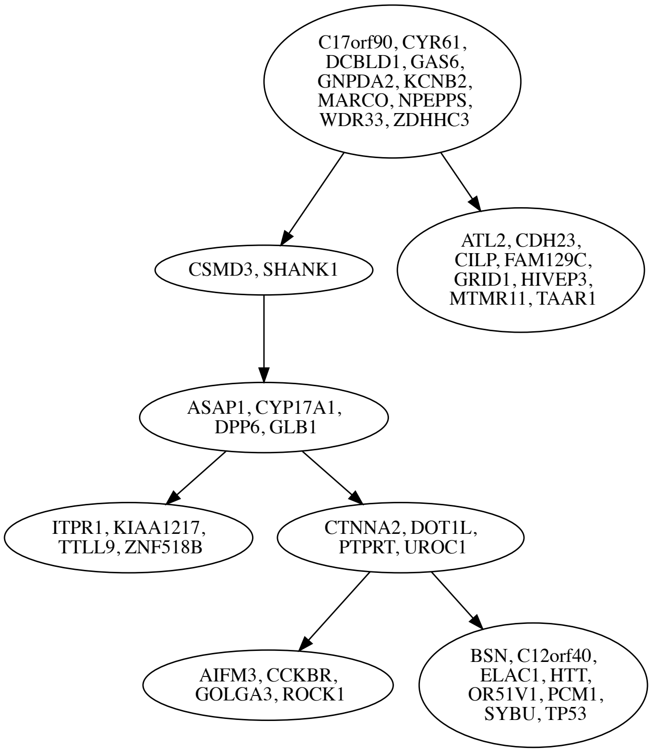

(a) Ours

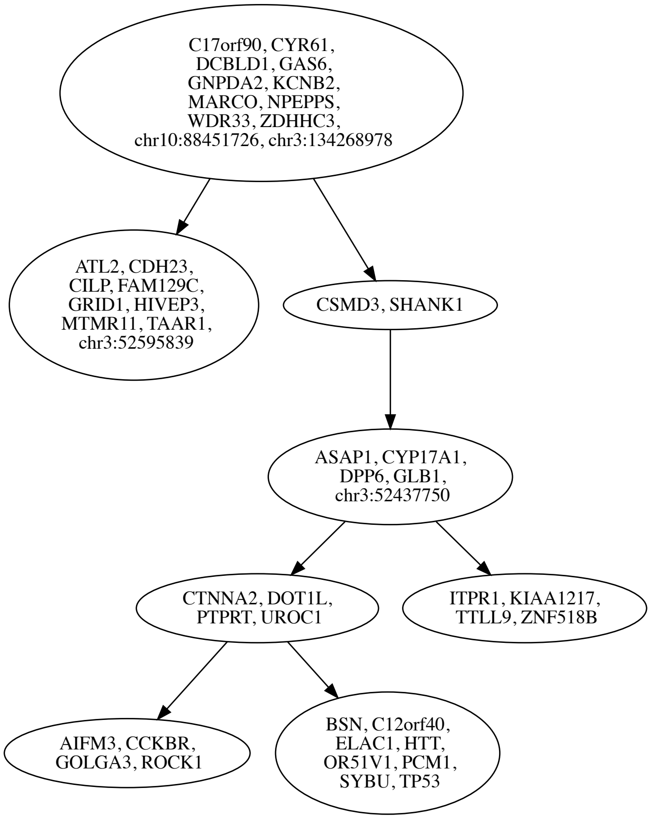

(b) LICHeE

Figure 9: RMH002

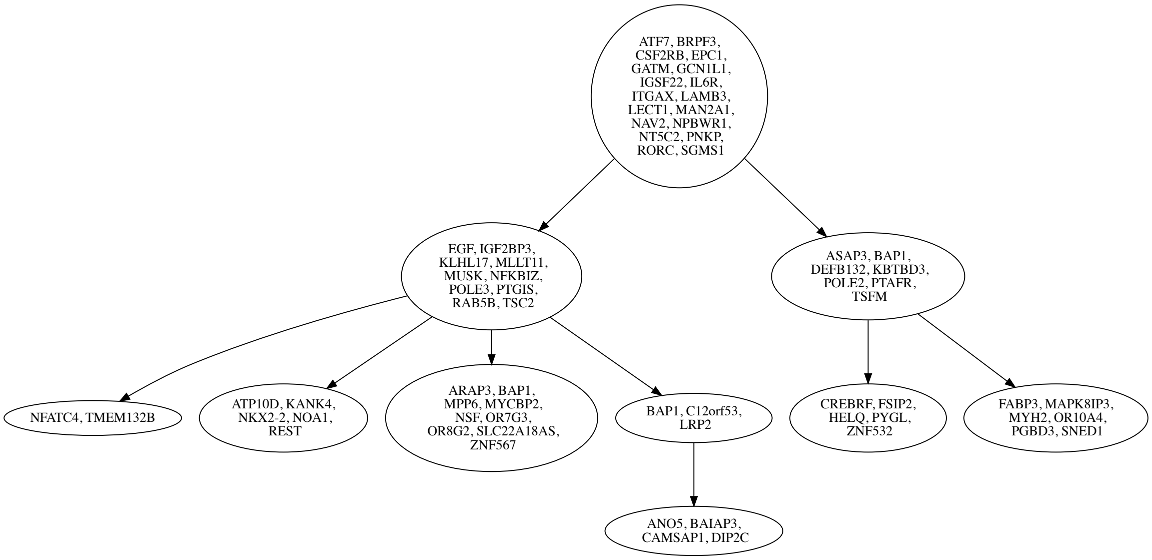

(a) Ours

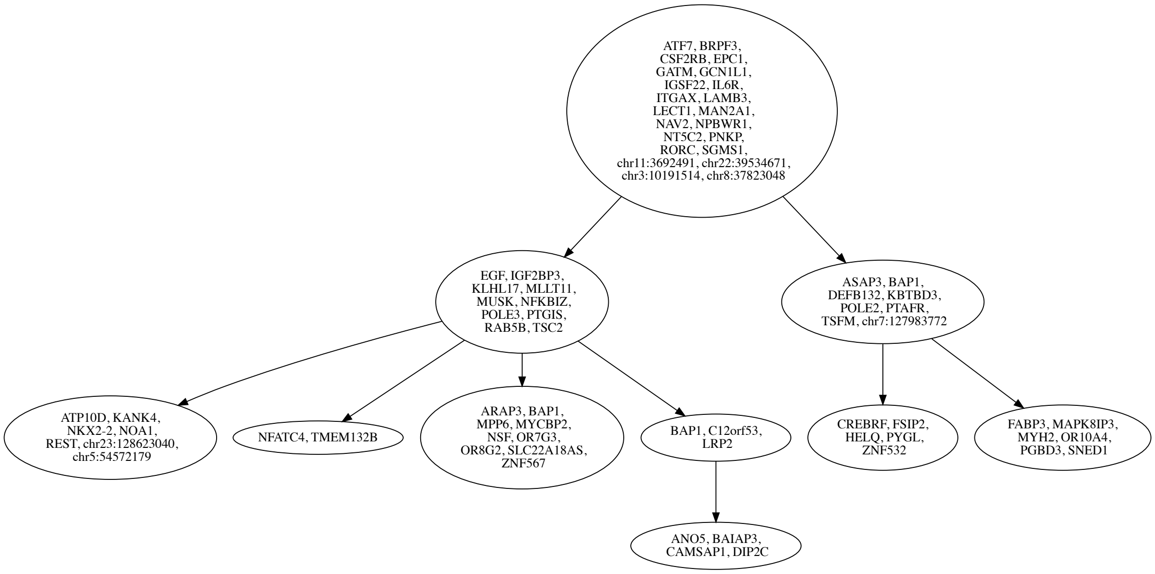

(b) LICHeE

Figure 10: RMH008
