# Supplementary material for: Parameter, noise, and tree topology effects in tumor phylogeny inference
Source: BMC Med Genomics. 2019 Dec 23;12(Suppl 10):184. doi: 10.1186/s12920-019-0626-0 (PMC6927103; doi:10.1186/s12920-019-0626-0)
Supplement: Supplementary file 3 — Additional file 3 Joint effect of clone count and sum condition relaxation. This PDF file contains plots showing the interaction effects of the clone count n and the sum condition relaxation parameter ε. [file 12920_2019_626_MOESM3_ESM.pdf]

# Joint Effect of Clone Count and Sum Condition Relaxation

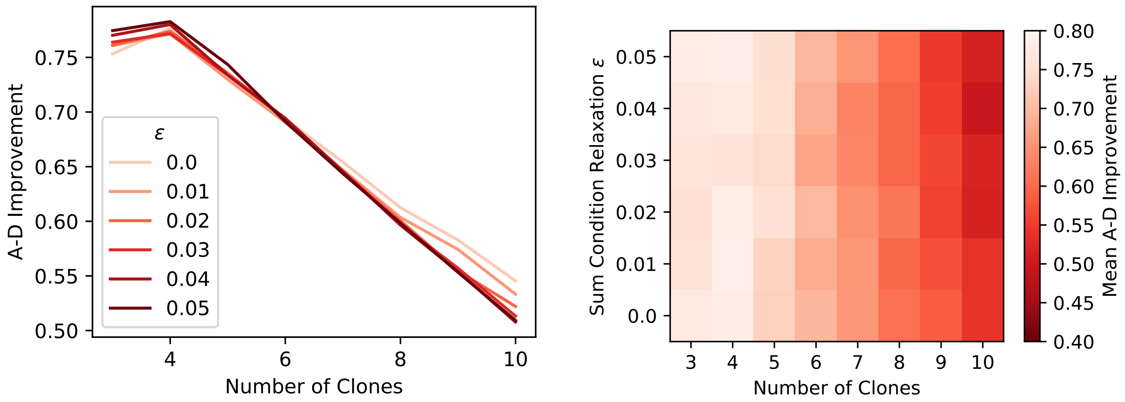


Figure 1: Two visualizations of the interaction effects of clone count and the sum condition relaxation parameter $\varepsilon$. Both plots show the same data generated from 10000 trials of the approximate ancestry graph method. Notice that higher values of $\varepsilon$ negatively impact solution quality when there are more than 6 clones, with a stronger effect at higher clone counts. Intriguingly, higher $\varepsilon$ appears to improve mean solution quality when $n < 6$. This may be related to the improved performance of the approximate ancestry graph for small $n$.
